# A Power Compensation Strategy for Achieving Homogeneous Microstructures for 4D Printing Shape-Adaptive PNIPAM Hydrogels: Out-of-Plane Variations

**DOI:** 10.3390/gels8120828

**Published:** 2022-12-15

**Authors:** Liyuan Tan, Hyunjin Lee, Li Fang, David J. Cappelleri

**Affiliations:** 1 School of Mechanical Engineering, Purdue University, West Lafayette, IN 47907, USA; 2 Weldon School of Biomedical Engineering, West Lafayette, IN 47907, USA

**Keywords:** hydrogel, PNIPAM, polymerization, 4D printing, homogeneous, deformation

## Abstract

In the last decade, 3D printing has attracted significant attention and has resulted in benefits to many research areas. Advances in 3D printing with smart materials at the microscale, such as hydrogels and liquid crystalline polymers, have enabled 4D printing and various applications in microrobots, micro-actuators, and tissue engineering. However, the material absorption of the laser power and the aberrations of the laser light spot will introduce a decay in the polymerization degree along the height direction, and the solution to this problem has not been reported yet. In this paper, a compensation strategy for the laser power is proposed to achieve homogeneous and high aspect ratio hydrogel structures at the microscale along the out-of-plane direction. Linear approximations for the power decay curve are adopted for height steps, discretizing the final high aspect ratio structures. The strategy is achieved experimentally with hydrogel structures fabricated by two-photon polymerization. Moreover, characterizations have been conducted to verify the homogeneity of the printed microstructures. Finally, the saturation of material property is investigated by an indirect 3D deformation method. The proposed strategy is proved to be effective and can be explored for other hydrogel materials showing significant deformation. Furthermore, the strategy for out-of-plane variations provides a critical technique to achieve 4D-printed homogeneous shape-adaptive hydrogels for further applications.

## 1. Introduction

3D printing techniques have been significantly upgraded and evolved to 4D printing through the use of smart materials over the past decade [1]. Structures achieved by these smart materials are able to deform under different stimuli such as, temperature, light, and pH values [2,3,4]. Recently, 4D printing has been applied to achieve microscale objects, especially with hydrogels or liquid crystalline polymers. Achieving 4D printing at the microscale opens up opportunities for the improvement of many applications [5]. For example, pyramid-like micro-structures and scaffolds are printed for bio-sensing and tissue engineering [6]. Furthermore, the technique has been adopted in the field of microrobotics which enables the possibility of the next generation microrobots with advanced functionalities [7]. Moreover, the combination of microscale 4D printing with structural color designs enables the potential uses in nanophotonic [8] or microrobotic applications [9].

Two-photon polymerization is one of the most commonly used methods for achieving microscale polymeric structures, such as microneedle arrays [10] and microrobots [11,12], with a resolution up to sub-100 nm [13,14]. This method typically uses a femtosecond laser to render a non-linear absorption in a polymerizable absorbing resin. However, the resin is absorbing the laser energy when the laser is passing through even if the laser is not focused at the point [15]. In the meantime, the size of the laser beam is becoming larger as the laser passes through [16] which is a typical phenomenon due to the mismatch in refractive index [17,18]. Moreover, this refractive index mismatch will also introduce a focal shift which will result in a shape difference. Both the power decay and the increase of the beam radius will result in a significant reduction in the density of radicals. When this density is lower than the threshold, no polymerization will occur. Williams et al. previously demonstrated power compensation in direct laser writing of SU-8 photoresist [19]. However, this material is not environmentally responsive and therefore using a slightly higher or lower power will not show a great effect on the resulting structures as long as the dose is higher than the threshold. Recent applications of this method have resulted in many different types of microstructures. However, since hydrogels or liquid crystalline polymers behave differently with various photoinitiators, solvents, co-polymers, and so on, there are only general understandings of 4D printing. The properties of the achieved structures should be assessed individually and systematically in order to obtain the robust design of adaptive structures. More details on the many applications of 4D printing can be found in [5,7,20].

When designing a structure that can deform into a required shape, homogeneous material properties for specific regions are preferred so that the deformed shapes can be easily predicted by approaches such as the finite element method. Structures with gradient material properties may result in non-intuitive deformations that introduces design complexities when the required structures are three dimensional. Furthermore, typical deformations achieved by gradient structures such as gradient strips or films can also be realized by the corresponding bilayer structures [21]. Moreover, 4D printing typically shows a low printing speed, especially when it uses a laser for line-wise scanning. Therefore, it is of importance to achieve the corresponding material properties for simulations, before performing experiments [22], which requires homogeneous samples for testing, as well as verifying the deformations achieved via experiments. There is a lot of literature on the aberration and absorption of two-photon polymerization/absorption, separately [19,23,24,25,26]. However, the discussions on their effects on 4D printing have not been reported since there is not a clear boundary for the polymerization threshold, i.e., different powers above the threshold will give a different response with a different crosslinking density.

Poly-*N*-isopropylacrylamide (PNIPAM) is a hydrogel material widely used in shape morphing structures [27,28]. These structures include responsive dumbbells [29], grippers [30], springs [31], and even microscale structures such as helical-shaped microrobots [32], and microgrippers [33] achieved by traditional photolithography. Recently, this hydrogel has been used for fabricating micro-structures via 4D printing such as micro-flowers [34], micro-helices [35], micro-umbrellas [36], and micro-transformers [37]. In the meantime, this hydrogel has been reported with various properties when it is co-polymerized with other materials that provide PNIPAM with additional properties. For example, acrylic acid is commonly used as a co-polymer with PNIPAM making the hydrogel pH-responsive [38,39,40,41]. Moreover, introducing nanoparticles (e.g., gold and ferroferric oxide) into the PNIPAM polymer matrix will make them light-responsive through a photothermal effect when it is exposed to lights with a corresponding wavelength [42,43,44,45].

In this paper, we propose a strategy to compensate the power decay along the height (out-of-plane) direction in order to achieve homogeneous and high aspect ratio micro-structures fabricated by a PNIPAM hydrogel. The strategy uses a piece-wise linear approximation for the compensation of the power drop. The strategy is calibrated with calibration prints with limited power drop which are compared with the resulting swelling for each step. The calibration prints are stimulated with different solvents to achieve swelling differences between different powers. Some example micro-structures have been printed based on the proposed strategy. Finally, several tests have been performed to characterize the printed structures. The proposed method can be readily adopted for other responsive hydrogel materials used for 4D printing with a significantly observable deformation. The results discussed in this paper on the compensation along the out-of-plane direction provides fundamental knowledge for achieving homogeneous structures to enable the robust design of adaptive structures for many applications.

## 2. Results and Discussion

### 2.1. Overview of the Calibration Process

**Figure 1:**
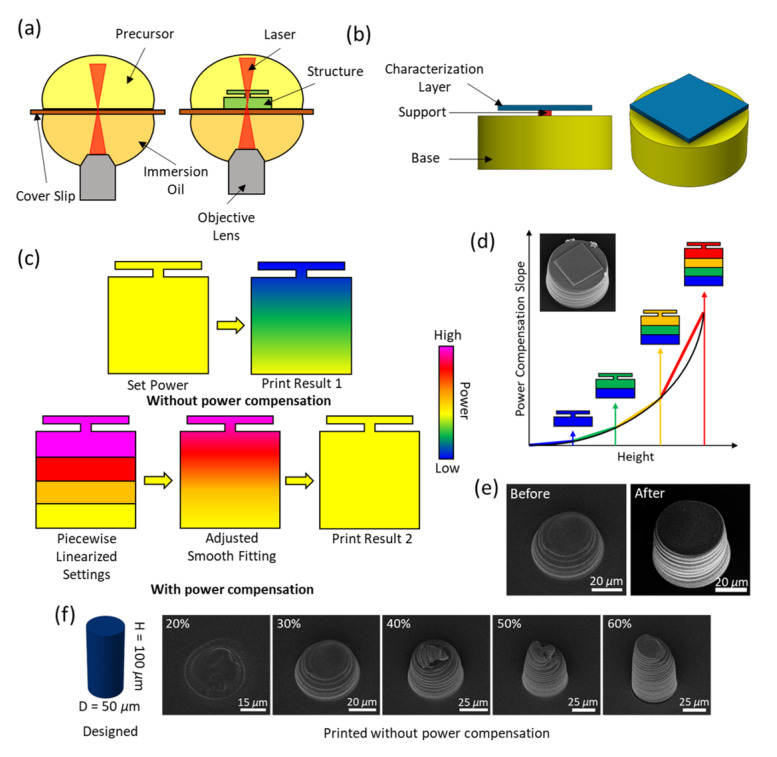
Schematic of polymerization decay in TPP with the oil-immersion mode. (**a**) Schematic of the two-photon polymerization process with the oil-immersion mode. (**b**) Model design for the characterization study of the power decay. (**c**) Route from the original sample to the compensated one. (**d**) Step-wise power compensation using a piece-wise linear slope from a gradually increased height. (**e**) 100 μm-tall pillar printed with an initial power of 30% before and after power compensation. The view of the images is tilted for 30${}^{\circ }$. (**f**) A cylinder printed with different powers without power compensation.

The calibration structures are printed using the oil-immersion mode as shown in Figure 1a. This mode is typically used for customized resins that will possibly be harmful to the objective lens. Moreover, for high absorbing resins, the intrinsic power decay from the lens to the focal point is significant. For example, the 63× lens used in the Nanoscribe TPP system (50 mW at 100% of power) has a working distance of 300 μm. Therefore, it is important to explore the oil-immersion printing mode and investigate the power decay along the printing direction. In this paper, the calibration structures, as shown in Figure 1b, are achieved by a characterization layer printed above a base connecting to a support structure. It is not a convinient or easy task to directly measure the laser power and beam diameter right after the laser passing through the already printed layers; therefore, the calibration process is realized by an indirect measurement of the equivalent power based on the deswelling ratio of the characterization layer. The deswelling ratio is defined as the ratio of the resulted length to the designed length.

**Figure 2:**
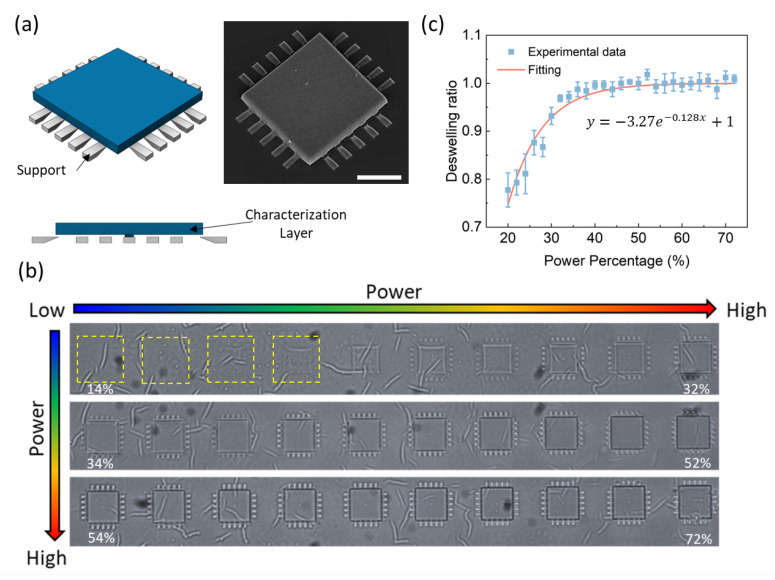
Standard prints for achieving calibration samples with limited power decay. (**a**) Suspended characterization layers with fin-like supports. (**b**) Calibration samples printed with different laser powers. The samples are printed with a power increment of 2% from 14% to 72% increased from left to right then from up to down. (**c**) Deswelling ratio data achieved from the standard prints and the fitting curve for calibration.

The calibration process is depicted in Figure 1c,d. Originally, the initial power (Set Power) used to achieve structures with a corresponding deswelling ratio is going to decay resulting in differences in the power and deswelling ratio (Print Result 1) along the height. Here, the equivalent power is used to represent the laser power achieving the required deswelling ratio. Not only the laser power decreases but the size of the focal spot of the laser is increasing. Since the decay of the equivalent laser power is not a linear process, we propose a piecewise-linear approximation of the decay of the equivalent power. Therefore, we calibrate the hydrogel material with a height of 100 μm with different deswelling ratios. Use the calibration process with a required deswelling ratio achieved by a 30% equivalent power, the calibration use 20 μm for each height step. A linear compensation (slope) of the initial power is used, and the initial power for a higher point is increased linearly based on the height of the point. With a parameter sweep of the slope, the calibration is performed by measuring the deswelling ratio of the characterization layer. The calibrated slope for the first step is then obtained via the fitting line using the deswelling ratio from the standard calibration prints (in Figure 2) with the corresponding equivalent power. The second step is also 20 μm on top of the first step but calibrated with a bottom part achieved by the first calibration. This process is repeated until it reaches 100 μm. Note: the step height may not be equal within that 100 μm. Then the piecewise-linear approximation (Piecewise Linearized Settings) is adjusted with semi-smooth non-linear fitting (Adjusted Smooth Fitting) to achieve a gradual change. Finally we expect to obtain high aspect ratio homogeneous structures (Print Result 2).

### 2.2. Standard Calibration Prints

The standard calibration prints are used to set a baseline for the calibration process. To obtain the standard prints, the characterization layer is printed via a model shown in Figure 2a. The standard prints are printed 1.4 μm above the adhesion layer supported with some pin-like structures to create suspended structures for characterization. The suspension heights of the standard prints are set to be as low as possible to minimize the power decay between the layer and the cover slip so that the power reduction can be ignored. The SU-8 adhesion layer is known to be transparent to NIR lights [46,47] so we assume the power reduction in the 500 nm adhesion layer is negligible since the wavelength of the laser for the TPP process is 780 nm in our system. Figure 2b shows the standard calibration prints with different laser powers for a consistent scanning speed of 8 mm/s in DI water. The side length of the characterization layer is set to be 25 μm. The power ranges from 14% to 72% with an increment of 2%. As can be seen from the images, no structures can be observed when the power is less then 20% while the structures printed over 40% show no difference. The measured deswelling ratios in DI water are plotted in Figure 2c showing a fast increasing trend before 40% but saturated trend to 1 after 50%. This is expected as the crosslinking degree is high for the structures achieved by a higher power, which results in a non-responsive structure [36].

### 2.3. Power Decay in Precursor and Printed Layers

**Figure 3:**
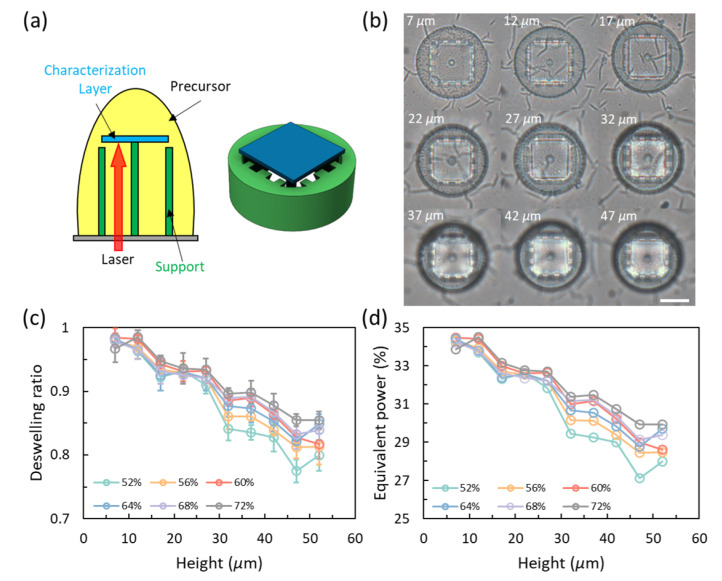
Power decay for penetrating the hydrogel precursor. (**a**) Schematic and model used for precursor penetration. (**b**) Deswelling of samples for penetration different depths with an initial power of 56%. Scale bar: 20 μm. (**c**) Deswelling ratios at different penetration depths with different initial laser powers. (**d**) The equivalent power converted from (**c**) based on the standard prints.

Some basic tests were performed to get a better understanding of the power decay when the laser passes through the precursor and printed layers. Figure 3a shows the schematic of the printed samples for the investigation of the power decay in the precursor. Peripheral and central supports are printed to support the characterization layer that is suspended without a base, generating a precursor environment for the laser to pass through a certain distance. Figure 3b presents the samples printed with different heights with a initial power of 56%. As can be seen in the figure, the size of the resulted characterization layers in DI water decreases with the height. Figure 3d gives the deswelling ratios of samples printed with different initial power ranging from 52% to 72% with an increment of 4%. Figure 3d is the corresponding equivalent power converted from Figure 3c based on the data of the standard prints in Figure 2c. Power decay data in the precursor for lower and higher powers than those shown in Figure 3 can be found in Figure S1 in the Supplementary Materials. Unlike the power decay of a laser beam passing through printed layers, the properties of the precursor is consistent along with height. In this case, all the curves in Figure 3d can fit into a commonly shared monotonous decaying curve.

**Figure 4:**
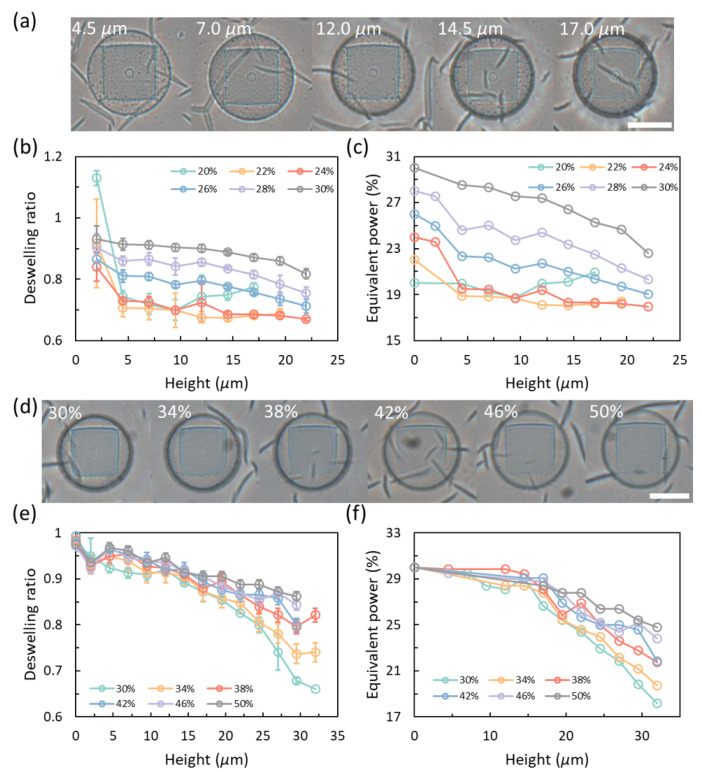
Power decay after penetrating printed layers. (**a**) Power decay along with height for penetrating layers achieved by the same initial laser power of 28%. (**b**) Deswelling data for different initial laser powers. (**c**) Equivalent power converted from the standard prints. (**d**) Power decay of the characterization layer achieved by an identical initial laser power of 30% but with a base printed by different initial powers. (**e**) Deswelling data for different initial laser powers. (**f**) Equivalent power converted from the standard prints. All scale bars: 25 μm.

The power decay of laser passing through printed layers is also investigated as shown in Figure 4. Figure 4a gives examples with different heights but printed with an initial power of 28%. Figure 4b,c show the data of the characterization layers achieved by the same model used for the calibration process (Figure 1b) with different initial power but without any power compensation. As can be seen from Figure 4c, the equivalent powers drop as the printing height is increased. With a higher initial power, the equivalent power drops linearly. However, when the initial power is less than 26%, the power will drop significantly at first and then the drop will slow down. A transition point is observed at power lower than 20% as the deswelling ratio is increased instead. At these powers lower than 20%, the hydrogel is crosslinked with a sparse network which absorbs water. However, if the power goes lower than this point, the matrix may not be formed. Figure 4d gives examples with an identical height of 19.5 μm but the bases are printed with different initial powers. Figure 4e,f show the results achieved with a non-compensated base while all the characterization layers are printed with an identical set power of 30%. As can be seen from the plots, the transmittances of the printed layers with different powers are different. The laser passed through a base printed with a higher initial power shows less decay than those achieved by a lower power. The equivalent power data in Figure 4c,f is converted from Figure 4b,e based on the standard calibration prints provided in Figure 2c. More data about the power decay through printed layers can be found in Figures S2 and S3 in the Supplementary Materials.

### 2.4. Calibrations for Different Deswelling Ratios

**Figure 5:**
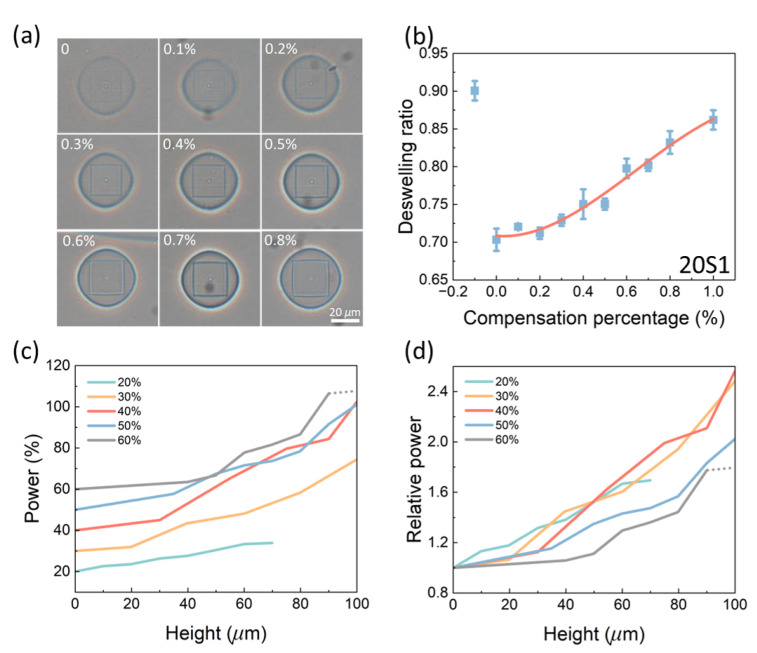
Power compensations obtained from the piecewise-linear approximation. (**a**) Samples compensated by different percentages with an initial laser power of 20%. (**b**) Deswelling ratios and fitting curve for Step 1 of the calibration. (**c**) Power compensation curves for different laser powers along the height. (**d**) Relative power compensations for different laser powers.

The calibration process is achieved by parameter sweeps for each step to find out the most appropriate slope for the corresponding step. Figure 5a,b give an example of the parameter sweeps for the first step with an initial laser power of 20%. The parameter sweep is set by different compensation percentages for each step. Using a different compensation percentage from the required one, the deswelling ratio will be different. The required compensation percentage is interpolated from the fitting curve of the deswelling ratios based on the standard value. As can be seen from Figure 5a, the square characterization layer is becoming larger as the compensation percentage is going up. Comparing the fitting result from Figure 5b with the standard value for an initial power of 20% gives a compensation percentage of 0.4% for each layer from 0 to 10 μm of height. Figures S4–S8 show deswelling ratios for different compensation percentages for different steps starting at initial powers of 20% to 60%, respectively. Table 1, Table 2 and Table 3 provide the compensation percentages all the steps for different initial laser powers. The targeted height of the calibration is 100 μm; however, the achievable height is limited by strong in-plane variations, most likely due to the boundary or other optical effects, and the achievable laser power of the system. The calibration for 20% power ends at 70 μm because of the strong in-plane variations while the calibration for 60% ends at 90 μm due to the power limitation of the machine. The in-plane variations are found especially when the structures are higher than 50 μm. Increasing the diameter of the base can reduce the effects of the in-plane variations on the characterization layer; however, the increase of the diameter will result in a longer printing time. Moreover, increasing the diameter from 50 to 80 μm does not lead to a measurable characterization layer at the height of 80 μm.

The achieved power compensation curves are plotted in Figure 5c with the relative power with respect to the initial value in Figure 5d. The relative power is defined by the ratio of the current power to the initial power at the height of 0 μm. As can be seen in Figure 5c, the power increases slowly for initial values larger than 20% followed by a sharp increase in the second step. However, this sharp increase slows down in a later step. These two transition points could be the transition of the relative importance between the intensity decay and the light spot aberrations. At the beginning, the intensity decay is dominant as the aberrations of the light spot are low. These two effects are comparable between the two transition points. After the second transition point, the aberrations of the light spot become more significant. A similar pattern was previously found with the peak intensity for multi-photon printing of SU-8 photoresist [19]. The power compensation for laser power of 50% and 60% are also calibrated for reference. However, the deformation at these high powers is too small to be recognizable since deswelling ratios remained close to 1, as shown in Figure 2c. Moreover, the deswelling ratios with different powers are fitted by an exponential curve with a limit of 1 at infinite which gives a fitted value slightly smaller than the experimental result. Nevertheless, similar transitions can also be found for these high powers. It can be seen that the initial slope in Step 1 has a higher value when compared with the slopes in later steps. We suppose that the layers obtained by 20% of power are barely above the polymerization threshold and the layers have similar optical properties to the pure precursor, which has a lower transmittance.

### 2.5. Characterization of the Calibrated Prints

To verify the homogeneity of the printed high aspect ratio structures, some characterizations are performed for verification from different perspectives. After the first verification, the compensation curve along the height will be slightly adjusted to achieve a more accurate result with a further verification.

#### 2.5.1. Direct Fitting of the Piecewise-Linear Curve

**Figure 6:**
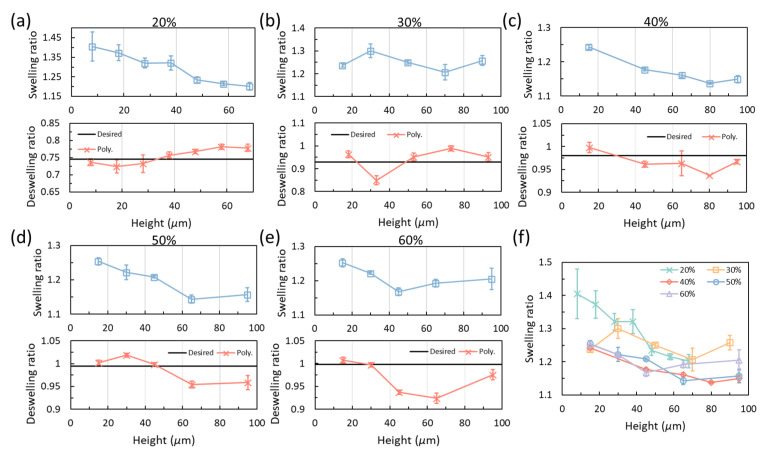
Verification prints using polynomial fitting of the piecewise-linear calibration results for different powers. (**a**) Results for 20% power. (**b**) Results for 30% power. (**c**) Results for 40% power. (**d**) Results for 50% power. (**e**) Results for 60% power. Upper plots in (**a**–**e**) are swelling ratios in alkaline solution and lower plots are in DI water. (**f**) Swelling ratios for different initial powers in alkaline solution.

To verify the calibration curves, the power compensation curves in Figure 5c are fitted by polynomials to achieve a semi-smooth change in the power by using two sets of polynomials for each curve. For example, the calibrated piecewise-linear curve for an initial power of 30% is separated by the point at the height of 40 μm where the slope becomes smaller. Then the powers in steps 1 and 2 are fitted by a fourth-order polynomial with a fixed intercept of 30%. The powers in steps 3 to 5 are fitted using a fifth-order polynomial with a starting power obtained from the previous fourth-order polynomial at 40 μm. An exponential fitting of the 30% curve has also been evaluated to prove the existence of the transition points as provided in Figure S9. Then the achieved polynomial curves of compensation power are applied with calibration models with heights different from those used to obtain the piecewise-linear slopes. Figure 6 gives the deswelling/swelling of the characterization layers printed by these curves in water and alkaline solution (pH = 12, sodium hydroxide in water). As can be seen from the figure, all the results for different initial powers obtained from the fitting curves deviate around the desired values when they are in water. For lower initial powers, i.e., 20% and 30%, with a deswelling ratio significantly lower than 1, the piecewise-linear calibration process is able to capture the desired values for all the heights, as can be seen in Figure 6a,b. For higher initial powers, the power is under-compensated when the prints are higher than 40 μm (Figure 6c–e). This is reasonable because the deswelling ratios for powers higher than 40% are too close to 1 and there are errors in measurements, thus, the calibration process may be capturing the deswelling ratio for 50% while the desired power is 60%. The deswelling ratios for 40% of power are only slightly off from the desired value. As a cross-verification of the calibrated results, the calibrated samples are also tested in alkaline solution. The printed hydrogel structures present swelling ratios with a resulting size larger than the designed size. However, it is found that the swelling ratio has an inverse trend when compared with the deswelling ratio in water for the cases with initial powers of 20% and 30%. When the initial power is higher than 40%, the trend shares a similar pattern. The measured swelling ratios in the alkaline solution are also plotted in Figure 6f for comparison. It can be seen that the swelling ratios from 40% to 60% are similar at all heights while the ratios for 20% and 30% are higher. Figure S10 gives the swelling ratios obtained from standard prints. All the measurable data are within the range of 1.20 to 1.28. The swelling ratio is generally increasing with power before and after 26%, with 26% being a minimum. However, the swelling ratios for power lower than 24% are not measurable without compensation as the entire structure becomes very similar to the precursor.

#### 2.5.2. From Direct Fitting to Adjusted Fitting

**Figure 7:**
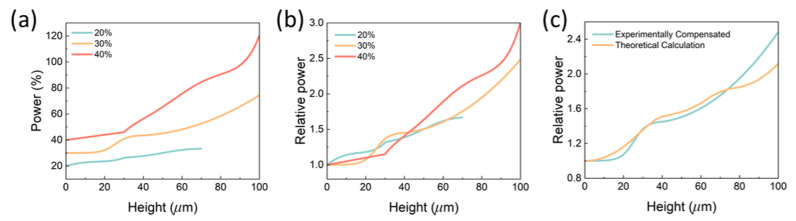
Adjusted fitting curves for lower initial powers. (**a**) Absolute power percentages at different heights after calibration. (**b**) Relative powers to the initial values. (**c**) Comparison of the adjusted compensation and the theoretical calculation.

Based on the results from the direct fitting, some adjustments have been made to modify the power compensation curves to obtain adjusted fittings based on the deswelling ratios in water: if the deswelling ratio is higher than the desired value, the power is lowered a little; and vice versa. This process is followed for the cases with initial powers of 20% and 30%. For the power of 40%, the adjustment is made by increasing relative power for 20%, assuming the crosslinking density has reached a peak at this point and further increasing the power will not affect the properties. However, it is indirectly proved that 40% of power provides a crosslinking density that keeps the deswelling ratio around 1 but the properties are not yet saturated. Figure 7a,b provide the adjusted curves and the corresponding relative power. Figure 7c shows a comparison between the adjusted fitting and the theoretical result for the power of 30%. The theoretical calculation is achieved based on the index mismatch between the immersion oil and the hydrogel resin (see Materials and Methods) which is also used for TPP process of SU-8 [19]. The theoretical calculation is in good agreement with the calibrated curve when the height is lower than around 70 μm. The deviation above 70 μm is probably because of the strong two-photon absorption that is not considered in the simplified model.

**Figure 8:**
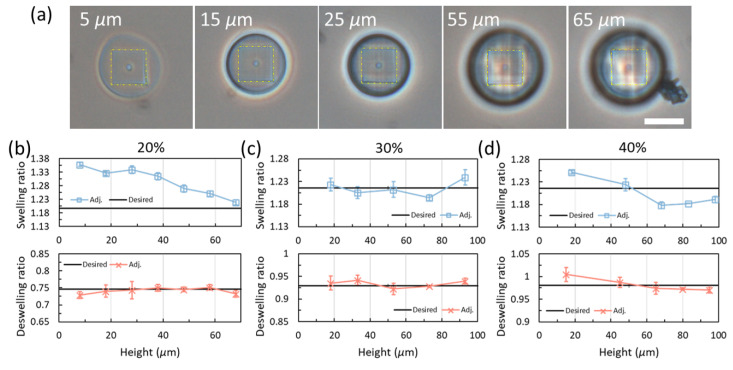
Deswelling and swelling tests with the adjusted fittings. (**a**) Samples printed with the adjusted curve for 20% with different heights. The yellow dash rectangles with an identical size mark out the sizes of the characterization layers. Scale bar: 25 μm. (**b**–**d**) Deswelling and swelling ratios of the samples printed with the adjusted curves in water and alkaline solution, respectively. (**b**) 20%. (**c**) 30%. (**d**) 40%.

The adjusted fittings are then applied to the same models used to verify the direct fittings. Figure 8a provides the deformed structures with different heights for 20% of power printed based on the adjusted fitting curve. The resulting sizes are very consistent in water and the deswelled characterization layers are highlighted by dashed rectangles in yellow. All the resulting deswelling ratios in water from 20% to 40% are well adjusted around the desired values as can be seen in Figure 8b. However, the desired value for 40% should be close to 1 based on experiments rather than the fitted value of 0.98. The adjusted fitting curves are tested in pH 12 alkaline solution as well showing decreasing trends for both powers of 20% and 40% while the swelling ratios for 30% are in reasonable good agreement with the desired value. Therefore, we assume that there are also in-plane variations of the power affecting the resulting deformations. Moreover, the measured swelling ratios in the alkaline solution can go lower than 1.2 while the ratios obtained from the standard prints are all higher than 1.2.

#### 2.5.3. Saturation of Crosslinking Density at Higher Powers

It is known that the crosslinking density is limited by the amount of the chemical components forming the polymer matrix, and once the applied exposure dose is higher than a saturation point, the crosslinking density will remain the same. Since there is no direct way to measure the material properties of the polymerized hydrogel as the power compensation is not calibrated, we adopt an indirect manner to prove the saturation by achieving a 2D to 3D deformation.

**Figure 9:**
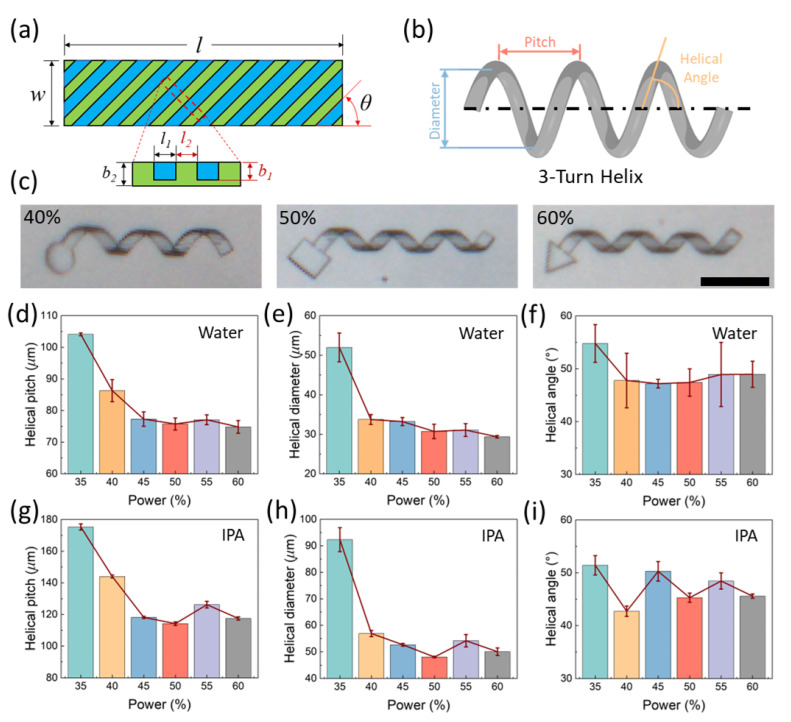
Indirect verification of the saturation at high power via 3D deformations. (**a**) Schematic of the modulating bilayer strip for 3D deformations. (**b**) Illustration of the helical parameters. (**c**) Deformed helical structures with different powers for the hard layer. Scale bar: 100 μm. (**d**–**f**) Changes of helical parameters in water with different powers for the hard layer. (**g**–**i**) Changes of helical parameters in IPA with different powers for the hard layer.

The schematic of the design of a 2D sample structure is given in Figure 9a. The design is previously adopted by Ji et al. to achieve deformations from 2D planar strips to 3D helical structures [35]. The planar strip consists of a modulation between soft (blue) and hard (green) regions. For structures immersed in water, the soft regions deswell while the hard regions remain the same, resulting in a deformation into 3D helical structures. However, the geometrical parameters of the deformed structures will be different if the stiffness of the hard region is different to the identical soft regions. The design parameters shown in Figure 9a for this paper are provided in the Experimental Section. The geometrical parameters are illustrated in Figure 9b with helical pitch, helical diameter, and helical angle. Here, a power of 30% is used for the soft regions while the hard regions are printed by a higher laser power from 35% to 60% with an increment of 5%. The resulting structures are shown in Figure 9c with examples for hard regions printed by 40%, 50%, and 60%, respectively. As can be seen from the examples, the 50% and 60% ones are similar, while the 40% print is not as compact as the previous two. The measured parameters for different powers are given in Figure 9d–f for water and Figure 9g–i in IPA. As shown in those figures, all the parameters decrease with an increasing hard region power and then decrease, reaching a saturation after the power of 45%. Therefore, we expect that the hydrogel matrix reaches a saturation on responsiveness in water at 40% but the mechanical property is saturated at around 45%.

#### 2.5.4. Chain Diameter and Porosity

**Figure 10:**
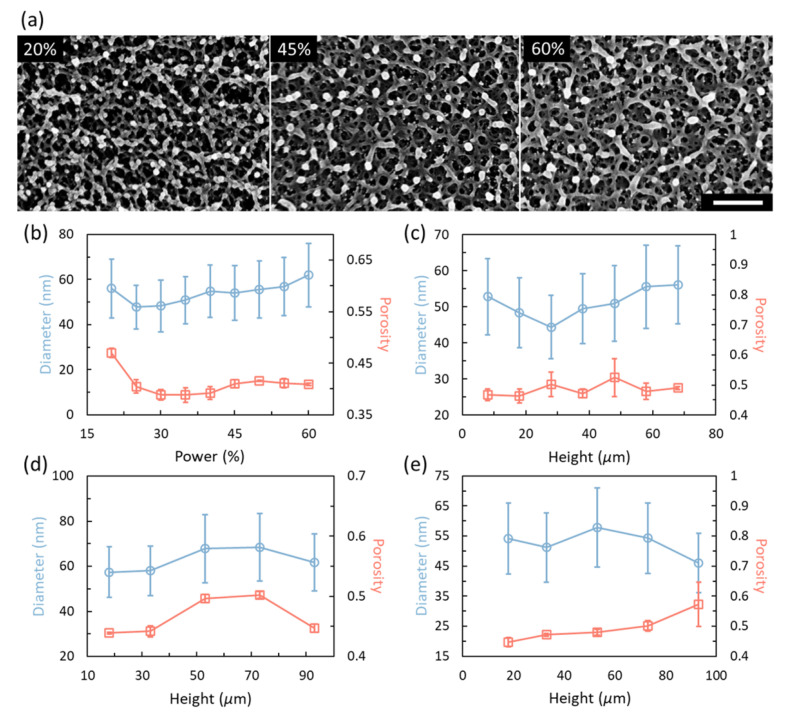
Nanostructure, chain diameter, and porosity of the 4D printed hydrogels. (**a**) Nanostructures of the hydrogel matrix from the standard prints obtained by critical point drying under ethanol. Scale bar: 500 nm. (**b**) Chain diameter and porosity of the standard calibration prints. (**c**) Chain diameter and porosity of samples calibrated for 20% power. (**d**) Chain diameter and porosity of samples calibrated for 30% power. (**e**) Chain diameter and porosity of samples calibrated for 40% power. All data points are obtained based on three samples. The error bars for blue curves are the standard deviation of the distribution of chain diameters.

The chain diameter (the diameter of the polymer chain forming the hydrogel matrix) and porosity of the standard prints and the calibrated samples are also investigated using scanning electron microscope (SEM) images. The average chain diameter is extracted by a customized MATLAB code based on a code provided for fiber diameter distribution [48,49] while the porosity is calculated as the ratio of the void area to the total area of the image. Figure 10a shows some sample SEM images of standard prints with different powers (also see Figure S12). As can be seen from the images, the hydrogel networks are generally very similar with a clear network after processing with ethanol. For comparison, Figure S11 shows SEM images for freeze-dried standard prints that were immersed in water. The strong deswelling at a lower power in water makes the network collapse and the network becomes clear when the power is above 40%. After 45%, the network structure becomes consistent in water. Figure 10b–d gives the obtained chain diameters and porosities of the standard prints and calibrated samples. However, since we are obtaining the information from 2D images of 3D structures and there are clusters in the hydrogel networks, the obtained results might be rough and only used for general understanding. Figure 10b gives the chain diameters and porosities of the standard prints with different powers. As shown in the plot, the average diameter decreases with the power until 25%. The value for powers from 40% to 55% are basically the same. However, the diameter at 20% is larger than the value at 25%. This is probably due to the power decay in the standard prints, which results in an equivalent power lower than 20%. We assume that the polymerization threshold of this hydrogel is a little bit below 18% power, and 20% is the power where the printed hydrogel presents a deswelling behavior in water. Printing with power between 18% and 20% will rather result in a swelling behavior, as can be seen in Figure 2. The change in porosity is more significant, as the porosity is generally decreasing with the increase of power. However, there is a slight increase after 40% of power and the value becomes steady after that. It should be noticed that the porosity is obtained in ethanol and the porosity is already reduced as the structure has deswelled (Figure S13). These steady values at powers higher than 45% also prove the saturation of the polymerization. The data obtained for calibration prints are less consistent as we suppose there is also an in-plane variation which will make the results different across the same surface. Figure 10c–e shows the results for powers of 20%, 30%, and 40%, respectively. In general, both the chain diameter and porosity of the samples vary about an average value along the height for 20% and 30%. The large difference in the results for 30% may come from the quality of the SEM images where the network inside structure is imaged with no difference with the surface network which introduces imaging processing errors. For the power of 40%, the diameter shows a decreasing trend while the porosity increases, especially when the height is above 73 μm. This decreasing/increasing trend could be the insufficiency of the compensation as the set power is getting to the limit. Furthermore, the strong in-plane variation may also introduce this problem as the standard deviation is large.

### 2.6. Discussion

The calibration process was completed based on the deswelling ratios in water for different laser powers. However, the calibrated results show some deviations when the structures are immersed in alkaline solution. These deviations might be introduced for the following reasons. First, the aberrations of the light spot will polymerize a larger region when the power is compensated as there are more regions that will be exposed by a dose higher than the threshold. The aberration is shown to have more significant effects on the axial direction of the light spot than the radial direction; therefore, an interpenetrating network may be formed when the light spot is large enough to affect the last printed layer [17]. Figure S14 gives the intensity profiles of the laser at different depths of penetration obtained by theoretical calculations corresponding to the theoretical curve in Figure 7. Second, the difference in the refractive index will result in a shift of the focal plane which may exaggerate the overlapping between layers (also see Figure S14). Moreover, there is a variation in the refractive index when the layer is polymerized by a different power (the higher the power, the smaller the mismatch of the refractive index), and the behavior of the light spot will be very complex. Figure S15 shows real-time images of the scanning lines during the printing with an initial power of 50% without power compensation. Third, we suppose there are also in-plane variations in each layer probably due to the edge effects from the shape of the structure. From the last structure of Figure 4d, the top of the base is deformed into an elliptical shape showing a strong anisotropic deformation along the laser scanning direction. The SEM images in Figure 3a of the non-compensated prints can also prove this in-plane variation. However, this obvious anisotropy is not found in the characterization layers. Nevertheless, this paper completed the calibration process for water-responsiveness which is experimentally taken based on the achieved deswelling ratio using different slopes that have the above effects into account. The process can be further refined by using a smaller steps with more samples for each data point. A better imaging method such as SEM can also be used for more accurate measurements of the deswelling ratios.

## 3. Conclusions

In this paper, we proposed a power compensation strategy to achieve homogeneous 4D-printed hydrogel structures along the out-of-plane direction. The strategy uses a piecewise-linear approximation to find out the power compensation needed to achieve homogeneous structures with a certain height. The slope for each piece of the approximation is obtained by interpolating the curve of deswelling ratios of the characterization layer with a set of calibrated slopes. The obtained piecewise-linear curve is then verified and adjusted to obtain a consistent swelling ratio for different heights. The calibration strategy gives a good power compensation result for hydrogels with a significant deswelling or swelling behavior, e.g., for 20% and 30%, while those without an obvious change are not able to be distinguished. The structures printed after power compensation show consistent deswelling behaviors as expected. Moreover, the saturation at powers higher than 45% is investigated with an indirect method with 3D deformations from planar to 3D structures. Finally, the calibrated prints are characterized by deswelling in water, swelling in alkaline solution, and SEM images. The compensation strategy provides a way for obtaining homogeneous structures along the out-of-plane direction.

## 4. Materials and Methods

### 4.1. Materials and Resin Preparations

The chemical-responsive poly-NIPAM (PNIPAM) hydrogel precursor is prepared similar to the process described in [36]. In brief, 1.6 g of *N*-isopropylacrylamide (NIPAM), 0.8 mL of acrylic acid (AAc), and 0.15 g polyvinylpyrrolidone (PVP) are dissolved in 1 mL of ethyl lactate (EL) followed by vigorous stirring for complete dissolution. Then 2.5 mL of the solution obatined above is mixed with 0.4 mL of dipentaerythritol pentaacrylate (DPEPA), 0.5 mL of triethanolamine (TEA), and 100 μL of 4,4’-bis(diethylamino)benzophenone/*N*,*N*-dimethylformamide (EMK/DMF) solution (1 to 4 weight ratio). The solution is magnetically stirred overnight to ensure complete dissolution. The refractive index of the prepared hydrogel precursor is measured to be 1.469 (Thermo Spectronic Refractometer).

### 4.2. Printing of the Hydrogel Micro-Structures for Calibration Prints

The hydrogel structures are printed by a commercial TPP system (Photonic Professional GT2, Nanoscribe GmbH) with the oil-immersion mode using a 63× objective lens. Before the printing, an adhesion layer of 500 nm of SU-8 is spin-coated on the glass cover slip and polymerized with UV light using SU-8 2000.5 which is diluted from SU-8 2010 with Cyclopentanone. Then the printing is performed on top of the adhesion layer with a scanning speed of 8 mm/s while the laser power is adjusted for the calibration process. The printed samples are then developed in IPA for 1 h followed by an equilibrium bath in water for another 1 h.

### 4.3. Printing of the 3D Helical Hydrogel Structures

The planar strips are printed with a consistent scanning speed of 8 mm/s. The total thickness of the strip is $b_{2}=5\;\mu$m with $b_{1}=4\;\mu$m for the thickness of the soft regions. The widths of the soft and hard regions are equally to be $l_{1}=l_{2}=4\;\mu$m. The total width and length of the strip are w=20μm and l=400μm, respectively. The modulating angle θ adopted here is 45${}^{\circ }$. The hard regions are printed first followed by the soft regions.

### 4.4. Sample Preparation for SEM

The printed samples are prepared using a critical point dryer (Autosamdri-931, tousimis) in the envrionment of pure ethanol and liquid carbon dioxide. Then the SEM images are taken by a Hitachi S-4800 SEM after coating with a gold/palladium (60:40) layer for around 20 nm.

### 4.5. Theoretical Calculation for Power Decay

The theoretical calculation for index mismatch power decay and aberration is based on the Gibson–Lanni model [50], with refractive index of objective immersion oil $n_{1}$ = 1.52, refractive index of substrate $n_{2}$ = 1.493, objective NA = 1.4, and λ = 780 nm. The relative power decay and intensity profiles are calculated along with the focal shift. More detail of the calculation can be found in the Supplementary Materials.

## Figures and Tables

**Table 1:** Calibration slopes for the equivalenet power of 20%.

	Step 1	Step 2	Step 3	Step 4	Step 5	Step 6	Step 7
Height ${}^{1}$	10	20	30	40	50	60	70
Slope ${}^{2}$	0.400	0.125	0.345	0.151	0.302	0.287	0.049

^1^ All height values are in unit of μm. ^2^ The slope is the percentage of power compensation for each layer comparing with the initial power for each step.

**Table 2:** Calibration slopes for the equivalent powers from 30% to 40%.

Equivalent Power		Step 1	Step 2	Step 3	Step 4	Step 5
30	Height ${}^{1}$	20	40	60	80	100
	Slope ${}^{2}$	0.098	0.545	0.16	0.319	0.413
40	Height ${}^{1}$	30	55	75	90	100
	Slope ${}^{2}$	0.125	0.541	0.331	0.118	0.646

^1^All height values are in unit of μm. ^2^ The slope is the percentage of power compensation for each layer comparing with the initial power for each step.

**Table 3:** Calibration slopes for the equivalent powers from 50% to 60%.

Equivalent Power		Step 1	Step 2	Step 3	Step 4	Step 5	Step 6	Step 7
50	Height ${}^{1}$	35	50	60	70	80	90	100
	Slope ${}^{2}$	0.132	0.525	0.379	0.091	0.194	0.496	0.313
60	Height ${}^{1}$	40	50	60	70	80	90	100
	Slope ${}^{2}$	0.044	0.584	0.488	0.152	0.183	0.676	-

^1^ All height values are in unit of μm. ^2^ The slope is the percentage of power compensation for each layer comparing with the initial power for each step.

## Data Availability

Not applicable.

## Supplementary Materials

The following supporting information can be downloaded at: https://www.mdpi.com/article/10.3390/gels8120828/s1, Figure S1: Power decay data for higher and lower initial power in precursor; Figure S2: Power decay along with height for penetrating layers achieved by the same initial laser power; Figure S3: Power decay of the characterization layer achieved by an identical initial laser power but with a base printed by different initial powers; Figure S4: Deswelling ratios for different compensation percentages for different steps for 20% of initial power; Figure S5: Deswelling ratios for different compensation percentages for different steps for 30% of initial power; Figure S6: Deswelling ratios for different compensation percentages for different steps for 40% of initial power; Figure S7: Deswelling ratios for different compensation percentages for different steps for 50% of initial power; Figure S8: Deswelling ratios for different compensation percentages for different steps for 60% of initial power; Figure S9: Deswelling ratios of samples printed with power compensation based on the exponential fitting with the piecewise-linear data; Figure S10: Swelling ratios of the standard prints in alkaline solution; Figure S11: Microstructures of the standard prints with different laser powers by freeze drying in DI water; Figure S12: Microstructures of the standard prints with different laser powers by critical point drying in ethanol; Figure S13: SEM images of the standard prints processed by critical point drying; Figure S14: Intensity profiles of the laser light at different penetration depths; Figure S15: Real-time scanning lines of the laser at different heights printing a model in Figure 1b with an initial power of 50%.

## References

[1] Khalid M.Y., Arif Z.U., Noroozi R., Zolfagharian A., Bodaghi M. (2022). 4D printing of shape memory polymer composites: A review on fabrication techniques, applications, and future perspectives. J. Manuf. Process..

[2] Liu X., Gao M., Chen J., Guo S., Zhu W., Bai L., Zhai W., Du H., Wu H., Yan C. (2022). Recent Advances in Stimuli-Responsive Shape-Morphing Hydrogels. Adv. Funct. Mater..

[3] Guan Z., Wang L., Bae J. (2022). Advances in 4D printing of liquid crystalline elastomers: Materials, techniques, and applications. Mater. Horizons.

[4] Kuang X., Roach D.J., Wu J., Hamel C.M., Ding Z., Wang T., Dunn M.L., Qi H.J. (2019). Advances in 4D Printing: Materials and Applications. Adv. Funct. Mater..

[5] Spiegel C.A., Hippler M., Münchinger A., Bastmeyer M., Barner-Kowollik C., Wegener M., Blasco E. (2020). 4D Printing at the Microscale. Adv. Funct. Mater..

[6] Liao C., Wuethrich A., Trau M. (2020). A material odyssey for 3D nano/microstructures: Two photon polymerization based nanolithography in bioapplications. Appl. Mater. Today.

[7] Adam G., Benouhiba A., Rabenorosoa K., Clévy C., Cappelleri D.J. (2021). 4D Printing: Enabling Technology for Microrobotics Applications. Adv. Intell. Syst..

[8] Zhang W., Wang H., Wang H., You J., Chan E., Ruan Q., Liu H., Yang J.K.W. (2022). 2.5D, 3D and 4D printing in nanophotonics—A progress report. Mater. Today Proc..

[9] Koepele C.A., Guix M., Bi C., Adam G., Cappelleri D.J. (2020). 3D-Printed Microrobots with Integrated Structural Color for Identification and Tracking. Adv. Intell. Syst..

[10] Faraji Rad Z., Prewett P.D., Davies G.J. (2021). High-resolution two-photon polymerization: The most versatile technique for the fabrication of microneedle arrays. Microsystems Nanoeng..

[11] Tottori S., Zhang L., Qiu F., Krawczyk K.K., Franco-Obregõn A., Nelson B.J. (2012). Magnetic helical micromachines: Fabrication, controlled swimming, and cargo transport. Adv. Mater..

[12] Yasa I.C., Tabak A.F., Yasa O., Ceylan H., Sitti M. (2019). 3D-Printed Microrobotic Transporters with Recapitulated Stem Cell Niche for Programmable and Active Cell Delivery. Adv. Funct. Mater..

[13] Zhou X., Hou Y., Lin J. (2015). A review on the processing accuracy of two-photon polymerization. AIP Adv..

[14] del Barrio J., Sánchez-Somolinos C. (2019). Light to Shape the Future: From Photolithography to 4D Printing. Adv. Opt. Mater..

[15] He G.S., Tan L.S., Zheng Q., Prasad P.N. (2008). Multiphoton absorbing materials: Molecular designs, characterizations, and applications. Chem. Rev..

[16] Correa D.S., De Boni L., Otuka A.J.G., Tribuzi V., Mendonça C.R. (2012). Two-Photon Polymerization Fabrication of Doped Microstructures.

[17] Hell S., Reiner G., Cremer C., Stelzer E.H. (1993). Aberrations in confocal fluorescence microscopy induced by mismatches in refractive index. J. Microsc..

[18] Diaspro A., Federici F., Robello M. (2002). Influence of refractive-index mismatch in high-resolution three-dimensional confocal microscopy. Appl. Opt..

[19] Williams H.E., Luo Z., Kuebler S.M. (2012). Effect of refractive index mismatch on multi-photon direct laser writing. Opt. Express.

[20] de Marco C., Alcântara C.C., Kim S., Briatico F., Kadioglu A., de Bernardis G., Chen X., Marano C., Nelson B.J., Pané S. (2019). Indirect 3D and 4D Printing of Soft Robotic Microstructures. Adv. Mater. Technol..

[21] Tan L., Davis A.C., Cappelleri D.J. (2021). Smart Polymers for Microscale Machines. Adv. Funct. Mater..

[22] Zhu H., He Y., Wang Y., Zhao Y., Jiang C. (2022). Mechanically-Guided 4D Printing of Magnetoresponsive Soft Materials across Different Length Scale. Adv. Intell. Syst..

[23] Cao C., Qiu Y., Guan L., Wei Z., Yang Z., Zhan L., Zhu D., Ding C., Shen X., Xia X. (2022). Dip-In Photoresist for Photoinhibited Two-Photon Lithography to Realize High-Precision Direct Laser Writing on Wafer. ACS Appl. Mater. Interfaces.

[24] Stichel T., Hecht B., Steenhusen S., Houbertz R., Sextl G. (2016). Two-photon polymerization setup enables experimental mapping and correction of spherical aberrations for improved macroscopic structure fabrication. Opt. Lett..

[25] Stichel T., Hecht B., Houbertz R., Sextl G. (2015). Compensation of spherical aberration influences for two-photon polymerization patterning of large 3D scaffolds. Appl. Phys. A Mater. Sci. Process..

[26] Horváth B., Ormos P., Kelemen L. (2017). Nearly aberration-free multiphoton polymerization into thick photoresist layers. Micromachines.

[27] Tang J., Yin Q., Qiao Y., Wang T. (2019). Shape Morphing of Hydrogels in Alternating Magnetic Field. ACS Appl. Mater. Interfaces.

[28] Yu R., Zhu L., Xia Y., Liu J., Liang J., Xu J., Wang B., Wang S. (2022). Octopus Inspired Hydrogel Actuator with Synergistic Structural Color and Shape Change. Adv. Mater. Interfaces.

[29] Han D., Lu Z., Chester S.A., Lee H. (2018). Micro 3D Printing of a Temperature-Responsive Hydrogel Using Projection Micro-Stereolithography. Sci. Rep..

[30] Zhang X., Xu X., Chen L., Zhang C., Liao L. (2020). Multi-responsive hydrogel actuator with photo-switchable color changing behaviors. Dye. Pigment..

[31] Yoshida K., Nakajima S., Kawano R., Onoe H. (2018). Spring-shaped stimuli-responsive hydrogel actuator with large deformation. Sens. Actuators B Chem..

[32] Huang H.W., Sakar M.S., Petruska A.J., Pané S., Nelson B.J. (2016). Soft micromachines with programmable motility and morphology. Nat. Commun..

[33] Fusco S., Sakar M.S., Kennedy S., Peters C., Bottani R., Starsich F., Mao A., Sotiriou G.A., Pané S., Pratsinis S.E. (2014). An integrated microrobotic platform for on-demand, targeted therapeutic interventions. Adv. Mater..

[34] Hu Y., Wang Z., Jin D., Zhang C., Sun R., Li Z., Hu K., Ni J., Cai Z., Pan D. (2019). Botanical-Inspired 4D Printing of Hydrogel at the Microscale. Adv. Funct. Mater..

[35] Ji S., Li X., Chen Q., Lv P., Duan H. (2021). Enhanced Locomotion of Shape Morphing Microrobots by Surface Coating. Adv. Intell. Syst..

[36] Jin D., Chen Q., Huang T.Y., Huang J., Zhang L., Duan H. (2019). Four-dimensional direct laser writing of reconfigurable compound micromachines. Mater. Today.

[37] Huang T., Huang H., Jin D.D., Chen Q.Y., Huang J.Y., Zhang L., Duan H.L. (2020). Four-dimensional micro-building blocks. Sci. Adv..

[38] Liu J., Jiang L., He S., Zhang J., Shao W. (2022). Recent progress in PNIPAM-based multi-responsive actuators: A mini-review. Chem. Eng. J..

[39] Sun X.F., Zeng Q., Wang H., Hao Y. (2019). Preparation and swelling behavior of pH/temperature responsive semi-IPN hydrogel based on carboxymethyl xylan and poly(N-isopropyl acrylamide). Cellulose.

[40] Zhang J., Chu L.Y., Li Y.K., Lee Y.M. (2007). Dual thermo- and pH-sensitive poly(N-isopropylacrylamide-co-acrylic acid) hydrogels with rapid response behaviors. Polymer.

[41] Park Y., Kim M., Chung H.J., Woo A.H., Noda I., Jung Y.M. (2021). The study of ph effects on phase transition of multi-stimuli responsive p(Nipaam-co-aac) hydrogel using 2d-cos. Polymers.

[42] Hauser A.W., Evans A.A., Na J.H., Hayward R.C. (2015). Photothermally Reprogrammable Buckling of Nanocomposite Gel Sheets. Angew. Chem..

[43] Zhou Y., Hauser A.W., Bende N.P., Kuzyk M.G., Hayward R.C. (2016). Waveguiding Microactuators Based on a Photothermally Responsive Nanocomposite Hydrogel. Adv. Funct. Mater..

[44] Lim D., Lee E., Kim H., Park S., Baek S., Yoon J. (2015). Multi stimuli-responsive hydrogel microfibers containing magnetite nanoparticles prepared using microcapillary devices. Soft Matter..

[45] Zhu C.H., Lu Y., Chen J.F., Yu S.H. (2014). Photothermal poly(N-isopropylacrylamide)/Fe3O4 nanocomposite hydrogel as a movable position heating source under remote control. Small.

[46] Ramirez J.C., Schianti J.N., Almeida M.G., Pavani A., Panepucci R.R., Hernandez-Figueroa H.E., Gabrielli L.H. (2017). Low-loss modified SU-8 waveguides by direct laser writing at 405 nm. Opt. Mater. Express.

[47] Parsi Sreenivas V.V., Winkler A., Harazim S., Schmidt O.G. (2018). Ultraviolet transmittance of SU-8 photoresist and its importance in multi-wavelength photolithography. J. Vac. Sci. Technol. B.

[48] Rabbani A., Salehi S. (2017). Dynamic modeling of the formation damage and mud cake deposition using filtration theories coupled with SEM image processing. J. Nat. Gas Sci. Eng..

[49] Ezeakacha C.P., Rabbani A., Salehi S., Ghalambor A. Integrated image processing and computational techniques to characterize formation damage. Proceedings of the SPE International Symposium on Formation Damage Control.

[50] Gibson S.F., Lanni F. (1992). Experimental test of an analytical model of aberration in an oil-immersion objective lens used in three-dimensional light microscopy. J. Opt. Soc. Am. A.

