# Supplementary material for: A Power Compensation Strategy for Achieving Homogeneous Microstructures for 4D Printing Shape-Adaptive PNIPAM Hydrogels: Out-of-Plane Variations
Source: Gels. 2022 Dec 15;8(12):828. doi: 10.3390/gels8120828 (PMC9778363; doi:10.3390/gels8120828)
Supplement: Supplementary file 1 [file gels-08-00828-s001.zip › gels-2065942-supplementary.pdf]

gels

MDPI

Supplementary Materials

Power Compensation Strategy for Achieving Homogeneous Microstructures for 4D Printing Shape-Adaptive PNIPAM Hydrogels: Out-of-Plane Variations

Liyuan Tan [1,†], Hyunjin Lee [2], Li Fang [2] and David J. Cappelleri [1,2,*]

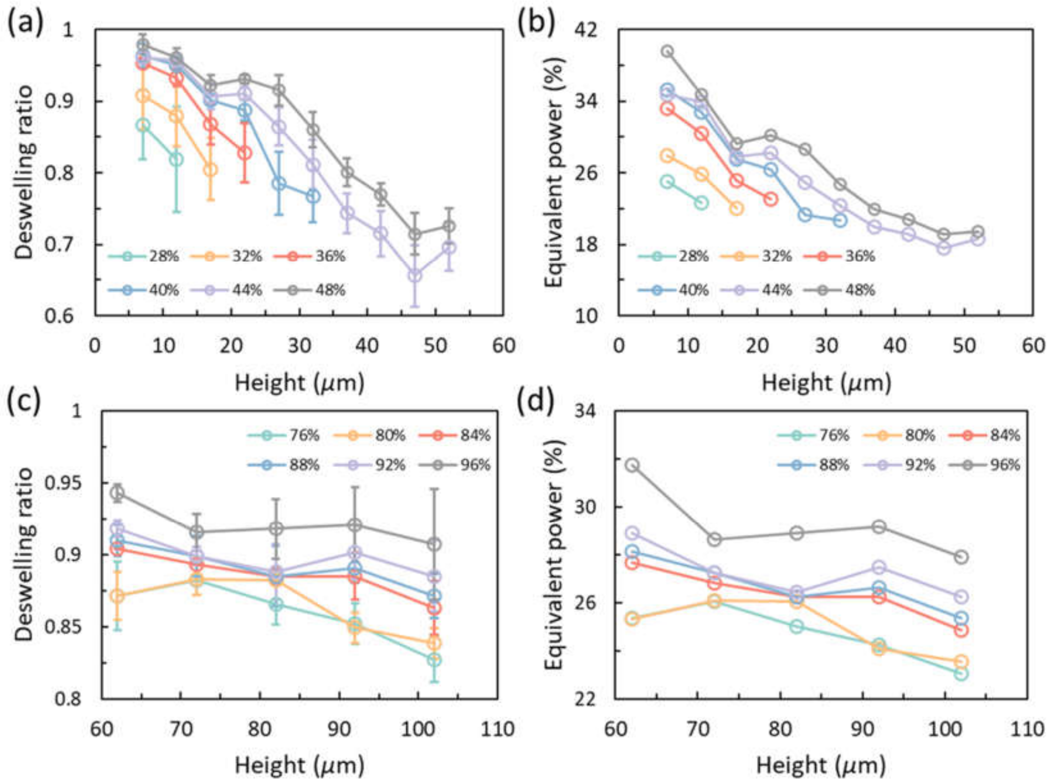

Figure S1. Power decay data for higher and lower initial power in precursor. (a) and (b) Deswelling ratio and equivalent power for powers from 28% to 48%. (c) and (d) Deswelling ratio and equivalent power for powers from 76% to 96%.

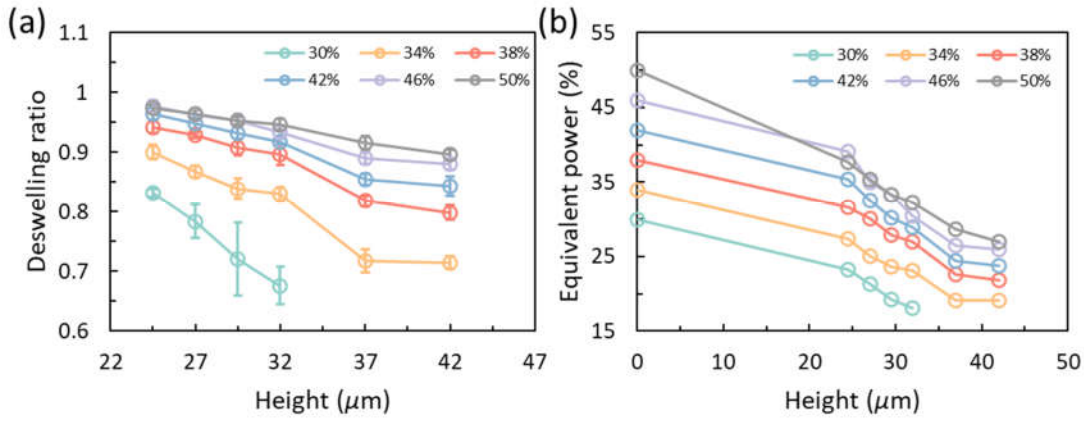

Figure S2. Power decay along with height for penetrating layers achieved by the same initial laser power. (a) and (b) Deswelling ratio and equivalent power for powers from 30% to 50%.

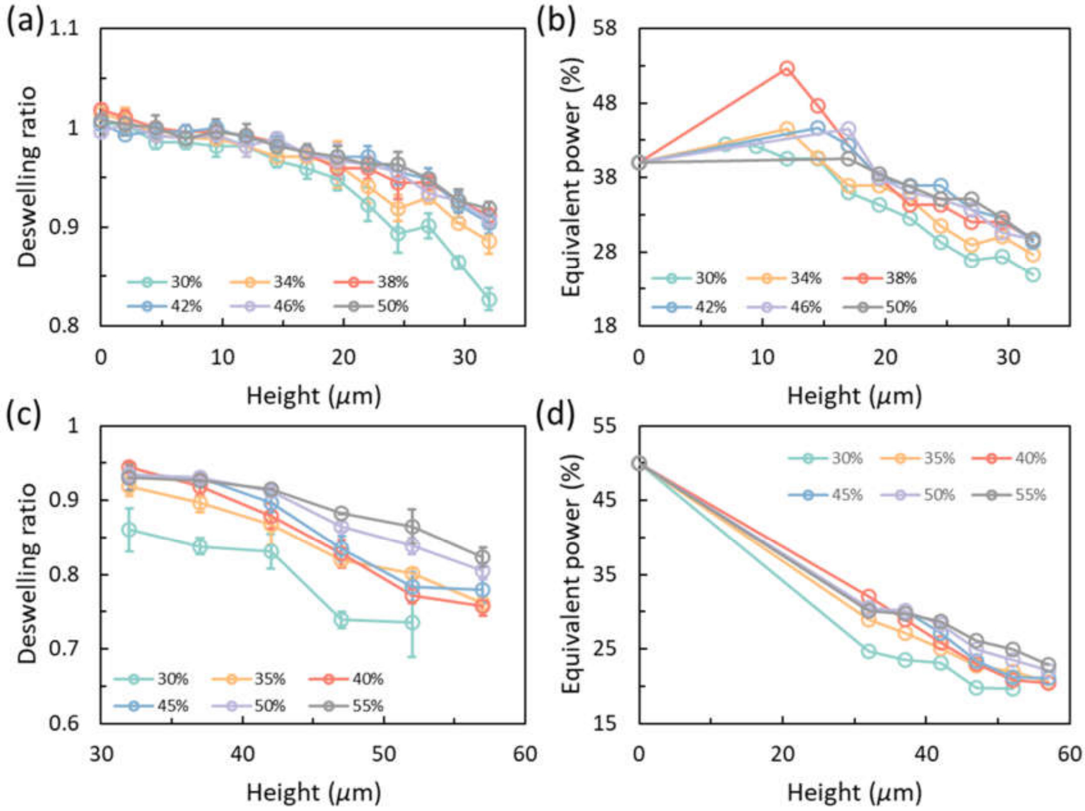

Figure S3. Power decay of the characterization layer achieved by an identical initial laser power but with a base printed by different initial powers. (a) and (b) Deswelling ratio and equivalent power for characterization layer with an initial power of 40%. (c) and (d) Deswelling ratio and equivalent power for characterization layer with an initial power of 50%. Legends are powers used to obtain the bases.

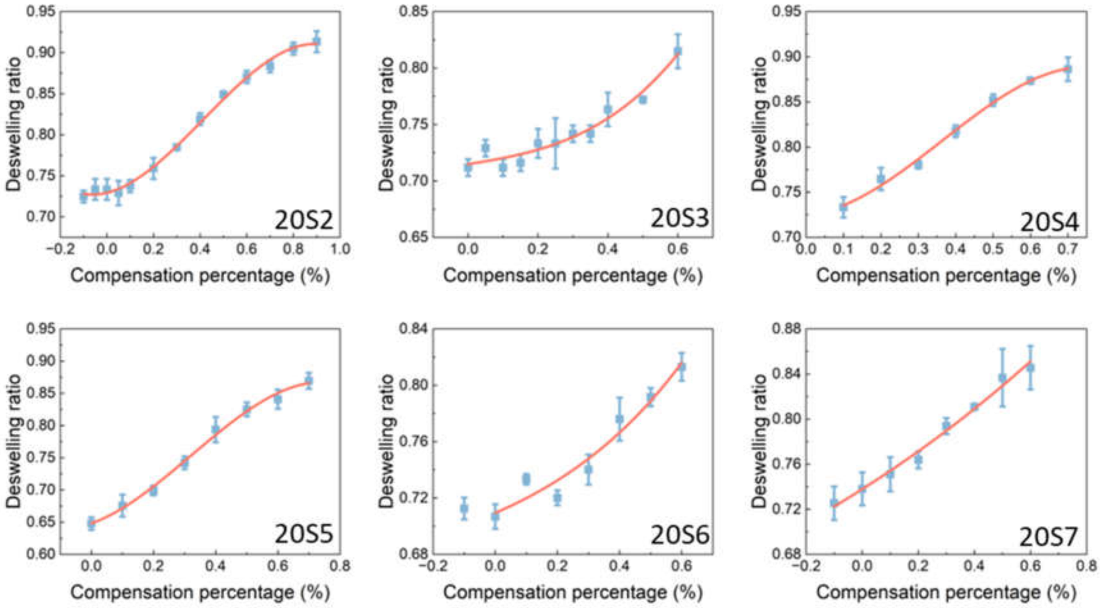

Figure S4. Deswelling ratios for different compensation percentages for different steps for 20% of initial power. The power and step number are marked on the bottom right of each plot. For example, "20S2" means step 2 for 20% of initial power.

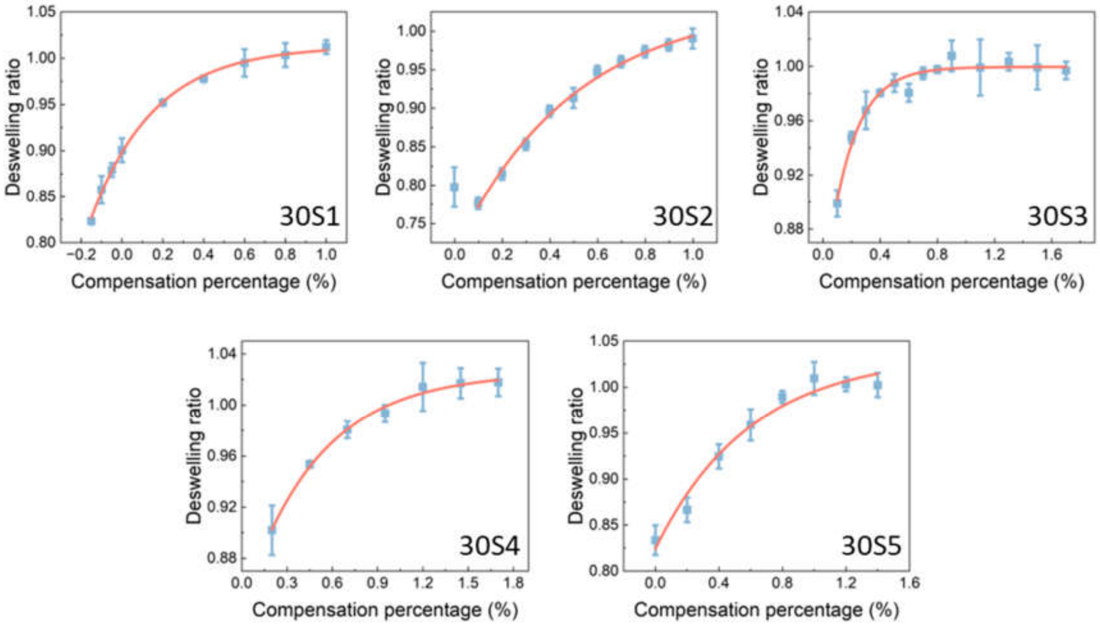

Figure S5. Deswelling ratios for different compensation percentages for different steps for 30% of initial power. The power and step number are marked on the bottom right of each plot. For example, "30S1" means step 1 for 30% of initial power.

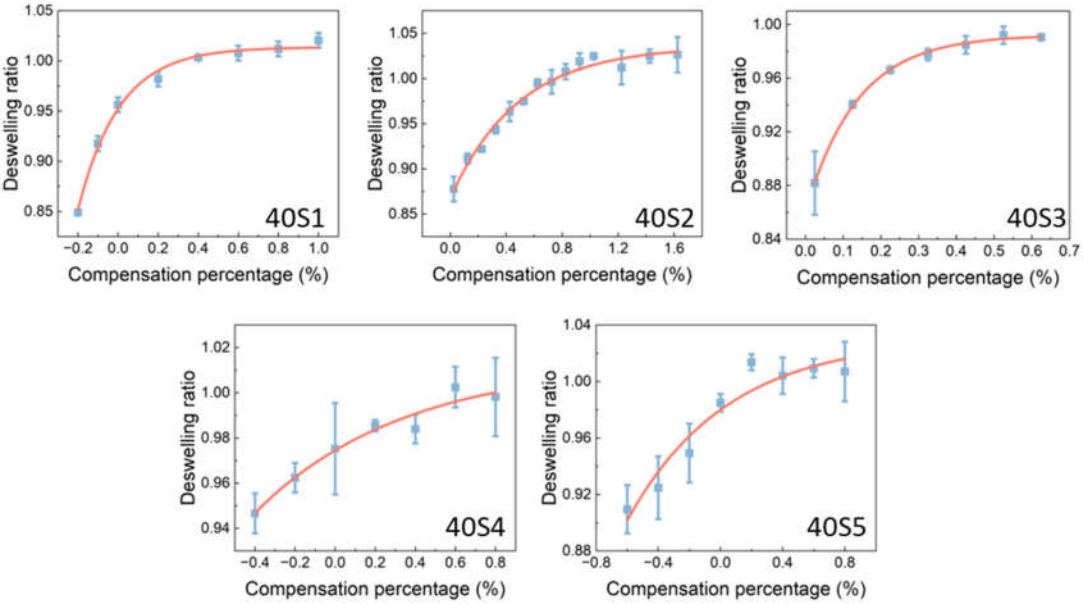

Figure S6. Deswelling ratios for different compensation percentages for different steps for 40% of initial power. The power and step number are marked on the bottom right of each plot. For example, "40S1" means step 1 for 40% of initial power.

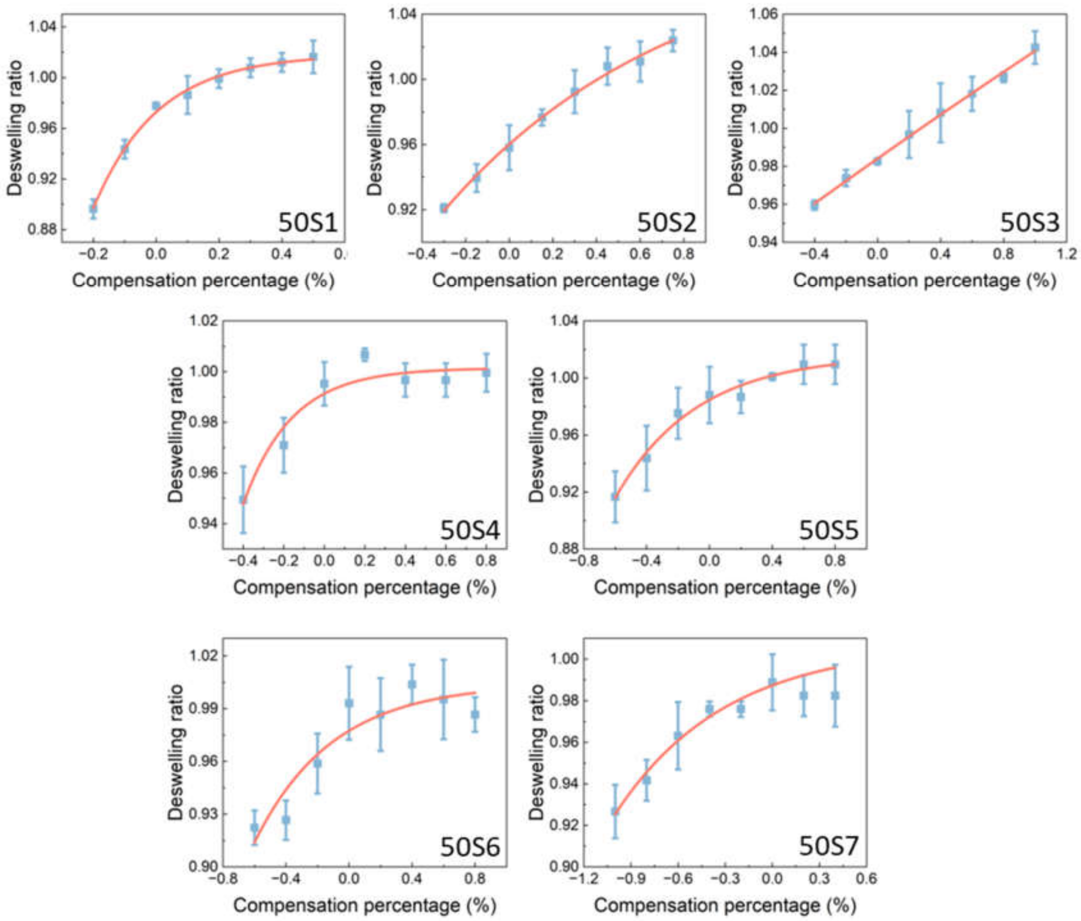

Figure S7. Deswelling ratios for different compensation percentages for different steps for 50% of initial power. The power and step number are marked on the bottom right of each plot. For example, “50S1” means step 1 for 50% of initial power.

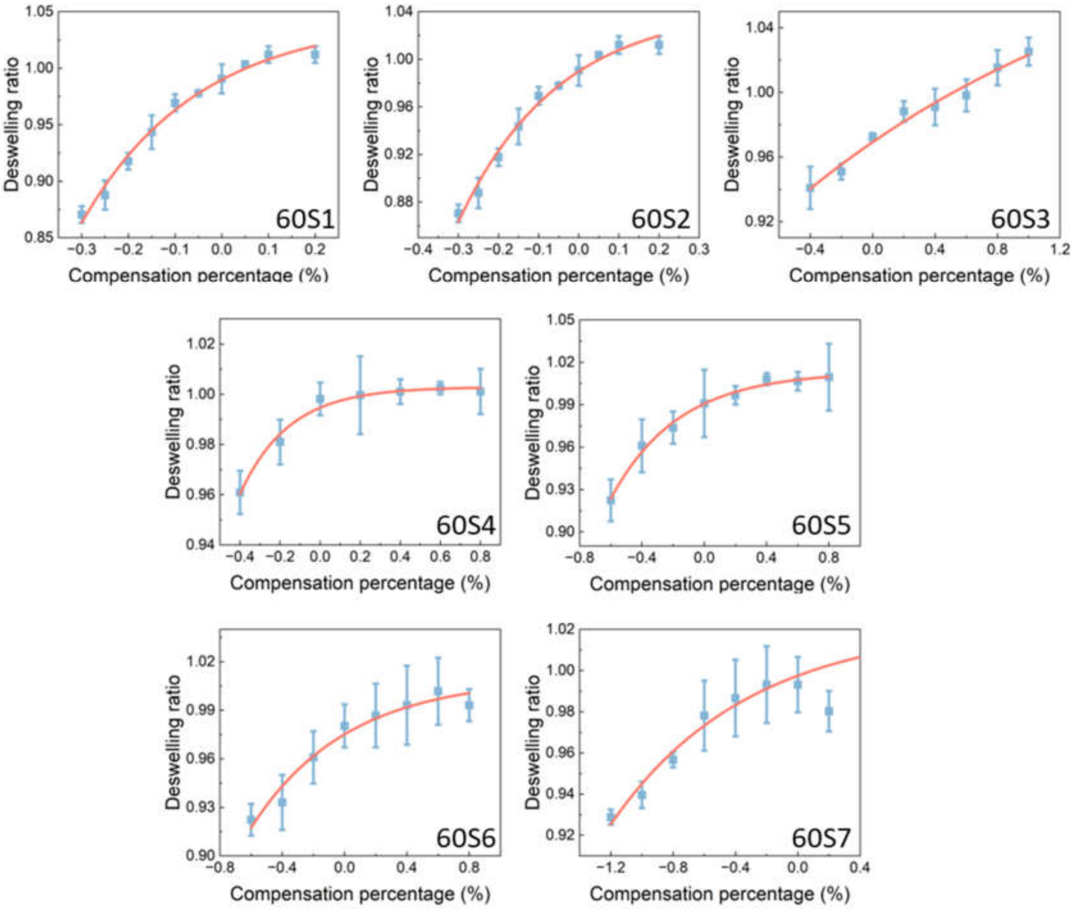

Figure S8. Deswelling ratios for different compensation percentages for different steps for 60% of initial power. The power and step number are marked on the bottom right of each plot. For example, "60S1" means step 1 for 60% of initial power.

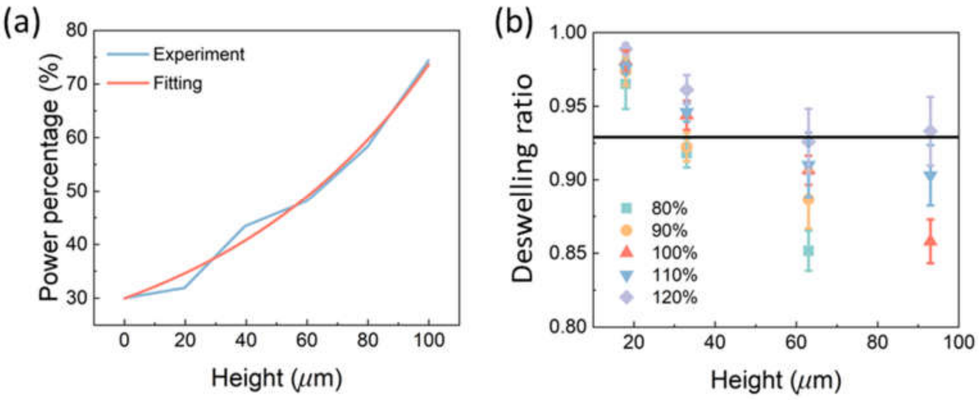

Figure S9. Deswelling ratios of samples printed with power compensation based on the exponential fitting with the piecewise-linear data. (a) The exponential fitting of the piecewise-linear curve. (b) Deswelling ratio of the samples based on the exponential fitting with adjustments with different percentages. The black line shows the desired deswelling ratio for the calibration.

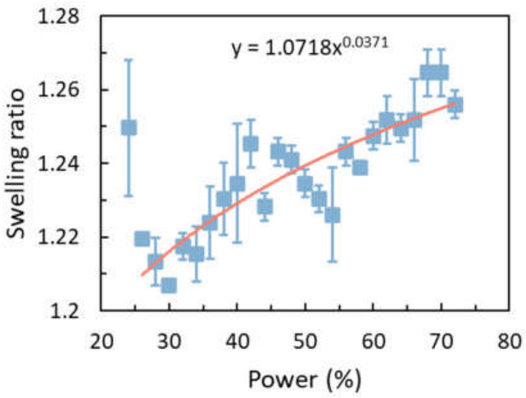

Figure S10. Swelling ratios of the standard prints in alkaline solution.

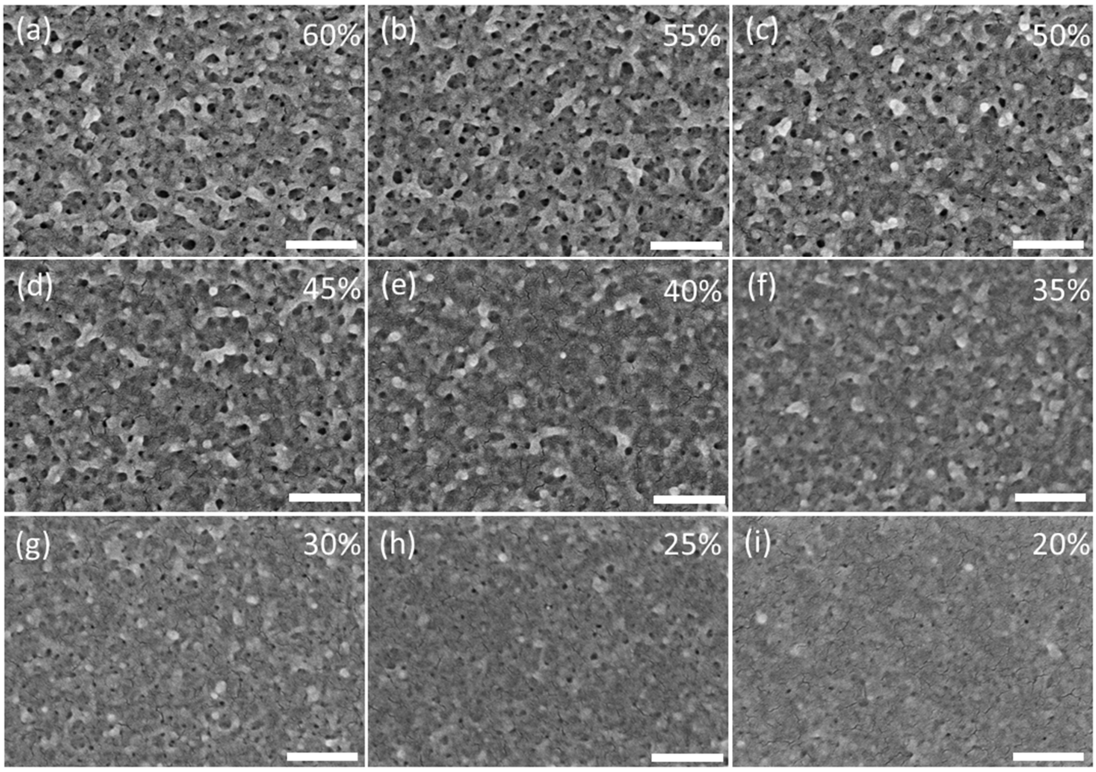

Figure S11. Microstructures of the standard prints with different laser powers by freeze drying in DI water. Scale bars: 500 nm.

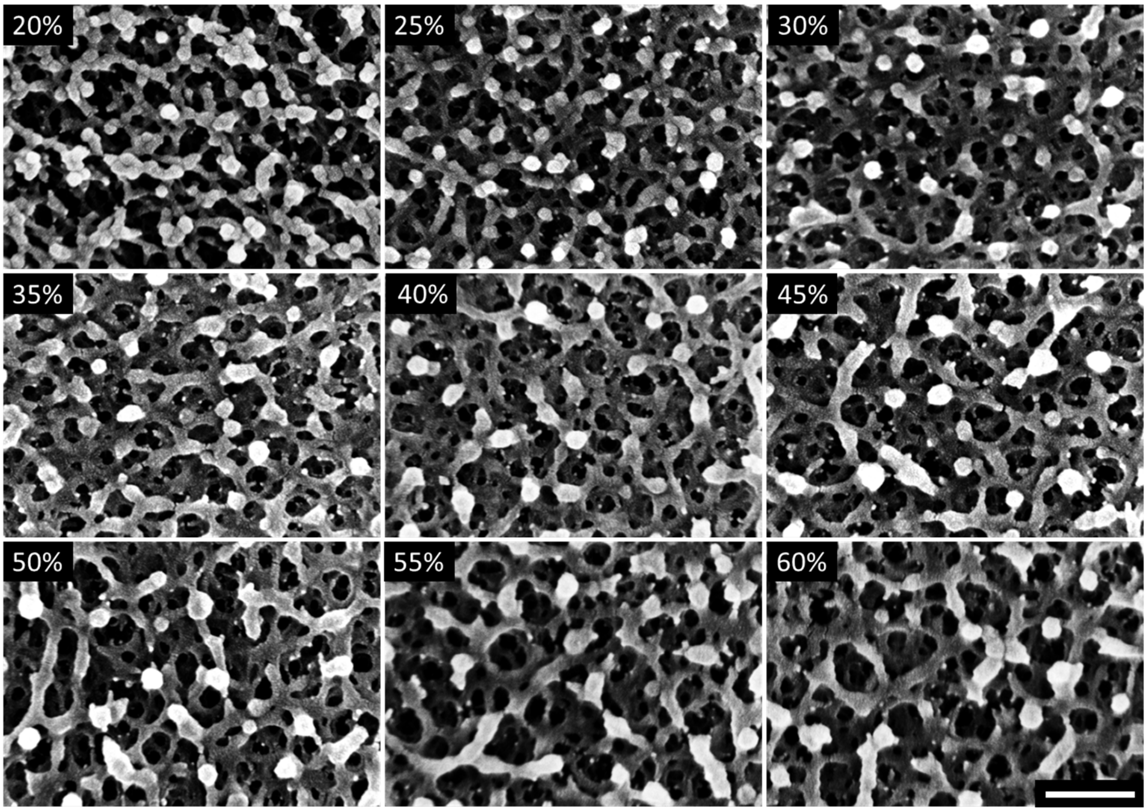

Figure S12. Microstructures of the standard prints with different laser powers by critical point drying in ethanol. Scale bar: 300 nm.

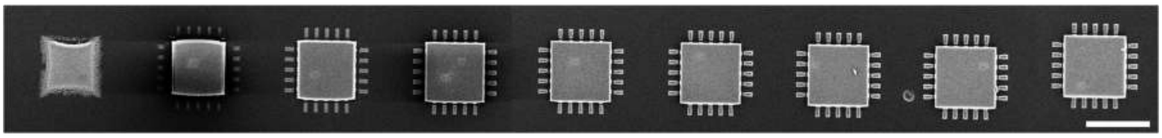

Figure S13. SEM images of the standard prints processed by critical point drying. The laser power is 20% to 60% from left to right with an increment of 5%. Scale bar. 25 μm.

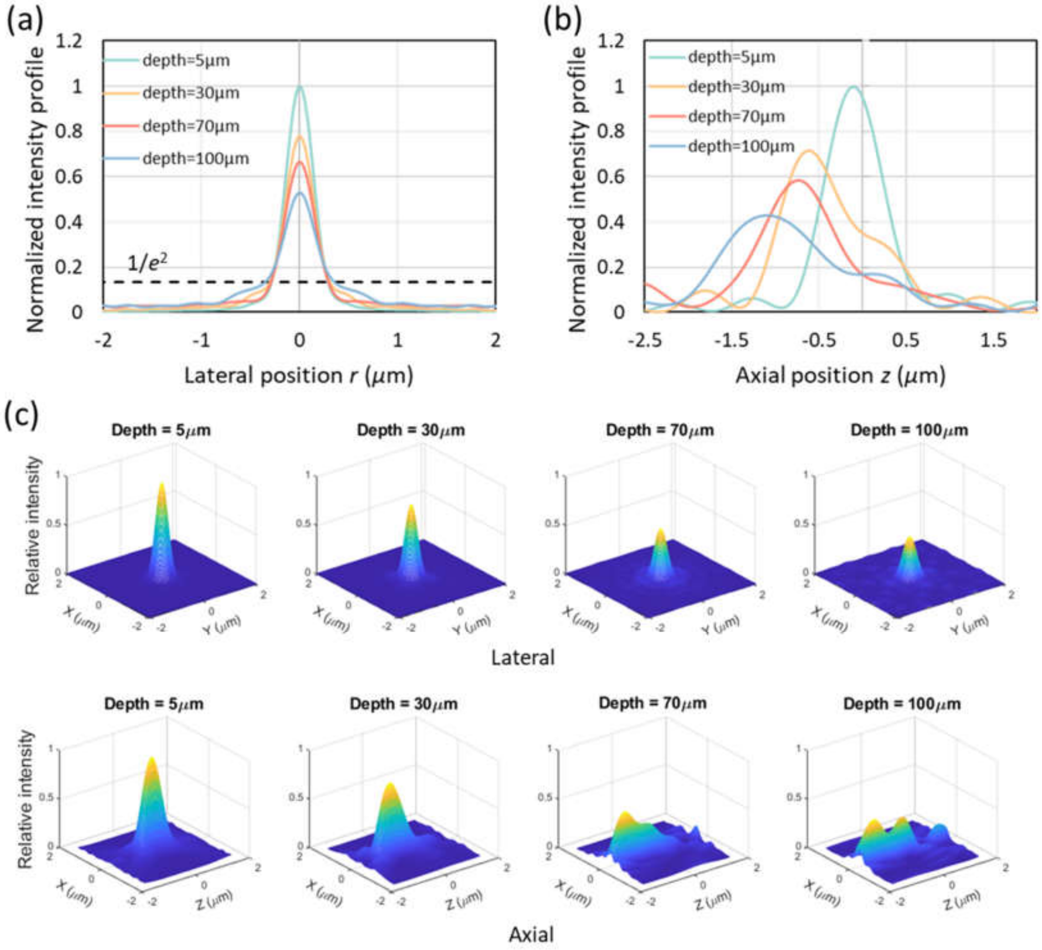

Figure S14. Intensity profiles of the laser light at different penetration depths. (a) Lateral profile. (b) Axial profile with focal shift. (c) 3D views of the intensity profile.

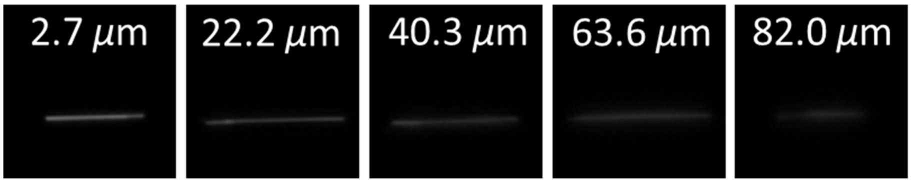

Figure S15. Real-time scanning lines of the laser at different heights printing a model in Figure 1b with an initial power of 50%.
